# Temporomandibular Disorders Related Pain among Sleep & Awake Bruxers: A Comparison among Sexes and Age

**DOI:** 10.3390/jcm12041364

**Published:** 2023-02-08

**Authors:** Ephraim Winocur, Mieszko Wieckiewicz

**Affiliations:** 1 Department of Oral Rehabilitation, The Maurice & Gabriela Goldschleger School of Dental Medicine, Tel Aviv University, Ramat Aviv, Tel Aviv 69978, Israel; 2 Department of Experimental Dentistry, Wroclaw Medical University, 50-425 Wroclaw, Poland

According to the current international consensus [1], bruxism should be divided into two circadian manifestations as awake bruxism (AB), which is “a masticatory muscle activity during wakefulness characterised by repetitive or sustained tooth contact and/or by bracing or thrusting of the mandible and is not a movement disorder in otherwise healthy individuals”, and sleep bruxism (SB), which is “a masticatory muscle activity during sleep that is characterised as rhythmic (phasic) or non-rhythmic (tonic) and is not a movement disorder or a sleep disorder in otherwise healthy individuals”. Bruxism is currently considered as a behavior and not as a parafunction or disorder in otherwise healthy individuals. It is a very complex phenomenon of central origin [2,3] that can affect the sleep structure [4], and can occur alone or with comorbidities such as obstructive sleep apnea (OSA), gastroesophageal reflux disease, insomnia, headache, orofacial pain, periodic limb movement, rapid eye movement behavior disorder, sleep epilepsy, anxiety disorders and depression [5,6,7].

Temporomandibular disorders (TMD) can be defined as a set of clinical problems involving the masticatory muscles, the temporomandibular joints, and related structures, and is related to alterations in the structure, function, or physiology of the masticatory system and may be associated with other systematic and comorbid medical conditions [8]. TMD is not a single disorder, rather it is the umbrella term that covers 37 entities in accordance with the taxonomy of Diagnostic Criteria for Temporomandibular Disorders (DC/TMD) [9]. Part of temporomandibular disorders are painful conditions that may co-occur with AB and SB [10,11,12,13,14,15,16].

Therefore, we encourage reading the important and novel articles published in this Special Issue, entitled “Temporomandibular Disorders Related Pain among Sleep & Awake Bruxers: A Comparison among Sexes and Age”. The articled are related, but not limited, to: the assessment of SB intensity in tobacco smokers and alcohol drinkers, assessment of masticatory muscle electromyographic (EMG) activity in both children diagnosed with pain-related TMD and AB and in children without TMD, symptoms of sleep masticatory muscle activity among women and their association to OSA, assessment of the effect of the current COVID-19 pandemic on the prevalence of bruxism, oral parafunctions and painful TMD among dental patients and to evaluate the influence of the pandemic on both sexes, testing the association between a single observation point self-report and ecological momentary assessment (EMA) of AB, cardiovascular implications of SB, and others. It is worth noting that readers can probably find, for the first time in the literature, research about the prevalence of TMD and bruxism among sex workers [17].
